# Immunohistochemical profiling of PD-L1 expression in neuroendocrine carcinoma of the uterine cervix

**DOI:** 10.3389/fimmu.2026.1933853

**Published:** 2026-09-22

**Authors:** Huang-Pin Shen, Ming-Yung Lee, Wan-Ru Chao, An-Chi Chen, Yi-Ju Lee, Chi-Kuan Chen, Chih-Ping Han

**Affiliations:** 1 Department of Obstetrics and Gynecology, Chung-Shan Medical University Hospital, Taichung, Taiwan; 2 Department of Obstetrics and Gynecology, Chung-Shan Medical University, Taichung, Taiwan; 3 Department of Data Science and Big Data Analytics, Providence University, Taichung, Taiwan; 4 Department of Pathology, Chung-Shan Medical University Hospital, Taichung, Taiwan; 5 Department of Pathology, Chung-Shan Medical University, Taichung, Taiwan; 6 Department of Biotechnology Medicine, MacKay Memorial Hospital, Taipei, Taiwan; 7 Department of Biotechnology Medicine, MacKay Medical University, Taipei, Taiwan

**Keywords:** 22C3 pharmDx, cervical neuroendocrine carcinoma, immune checkpoint inhibitor, immunohistochemistry, PD-L1

## Abstract

**Introduction:**

Neuroendocrine carcinoma (NEC) of the uterine cervix is a rare and aggressive malignancy for which disease-specific treatment strategies remain limited. Although immune checkpoint inhibitors (ICIs) have demonstrated activity in several cancers, PD-L1 expression in cervical NEC remains insufficiently characterized. This study descriptively evaluated PD-L1 expression patterns in cervical NEC using the PD-L1 IHC 22C3 pharmDx assay.

**Methods:**

Six surgically resected cervical NEC specimens from Taiwanese women treated between 2010 and 2024 were retrospectively analyzed. PD-L1 expression was assessed using the PD-L1 IHC 22C3 pharmDx assay. TPS, CPS, and IC scores were evaluated descriptively; TC was also recorded but was numerically identical to TPS in all cases and is therefore reported jointly as TPS/TC. Thresholds of TPS/TC ≥1%, CPS ≥1, and IC ≥5% were used solely for exploratory analyses and were not interpreted as validated clinical cutoffs for cervical NEC.

**Results:**

Tumor-cell PD-L1 expression was uncommon, with TPS/TC ≥1% observed in one case (16.7%). CPS ≥1 and IC ≥5% were each observed in three of six cases (50%). PD-L1 staining was more frequently observed in tumor-infiltrating immune cells than in tumor cells. One case demonstrated dual-compartment staining, with CPS 25, TPS/TC 3%, and IC 30%.

**Discussion:**

In this small cohort, PD-L1 expression was heterogeneous. Tumor-cell positivity by TPS/TC was uncommon and largely limited to a single case, whereas PD-L1 staining was more frequently observed in immune cells. CPS and IC provided complementary descriptive information on PD-L1 distribution; however, their biological relevance and predictive value remain unvalidated in cervical NEC. Larger studies integrating molecular profiling, functional immune characterization, and clinical outcome data are needed to determine the potential clinical utility of PD-L1 assessment in this rare malignancy.

## 1 Introduction

Neuroendocrine carcinoma (NEC) of the uterine cervix is a rare and highly aggressive malignancy, accounting for approximately 0.9–2.0% of invasive cervical cancers (1). Compared with squamous cell carcinoma and adenocarcinoma, cervical NEC is characterized by early lymphovascular invasion, higher rates of lymph node and distant metastasis, and poor prognosis across disease stages (1, 2). Because of its rarity, treatment is generally based on multimodal approaches and is often extrapolated from cervical cancer and neuroendocrine carcinoma protocols, including surgery, platinum-based chemotherapy, and radiotherapy (1–3). Despite multimodal treatment, recurrence is frequent, and survival remains poor, underscoring the need for novel systemic therapies (1–3).

Programmed death-ligand 1 (PD-L1, CD274/B7-H1) contributes to tumor immune evasion by engaging programmed death-1 (PD-1) receptors on activated T cells, thereby dampening cytotoxic antitumor immunity (4). ICIs targeting the PD-1/PD-L1 pathway, including pembrolizumab, nivolumab, atezolizumab, and durvalumab, have demonstrated clinical activity across multiple tumor types (5–7). These findings have generated interest in extending checkpoint blockade to rare, high-grade tumors such as cervical NEC, for which conventional treatment provides limited disease control.

PD-L1 immunohistochemistry (IHC) is an established biomarker-assessment approach for selecting patients for checkpoint inhibitor therapy in several tumor types (5–7). The PD-L1 IHC 22C3 pharmDx assay (Agilent Technologies) is indicated for cervical cancer, for which PD-L1 expression is assessed using a combined positive score (CPS) threshold of ≥1 (8–10). However, the clinical evidence and regulatory indication for cervical cancer were not specifically established for neuroendocrine histology. Accordingly, application of the 22C3 assay and its CPS threshold to cervical NEC should be regarded as investigational rather than as a histology-specific validated biomarker strategy.

Given the lack of biomarker strategies specifically validated for cervical NEC, PD-L1 assessment may provide useful information about the tumor immune microenvironment and may help generate hypotheses for future immunotherapy studies. This study performed immunohistochemical profiling of PD-L1 using the 22C3 pharmDx assay in six cases of primary NEC of the uterine cervix to characterize expression patterns and explore their potential biological relevance in this rare malignancy.

## 2 Materials and methods

### 2.1 Case selection

Six histologically confirmed primary NEC cases of the uterine cervix were retrospectively identified among Taiwanese women who underwent surgical resection at Chung Shan Medical University Hospital between 2010 and 2024. All specimens were formalin-fixed, paraffin-embedded (FFPE) tissue obtained by hysterectomy or tumor debulking; biopsy-only and cytology-only specimens were excluded. The study was approved by the Institutional Review Board (IRB) of Chung Shan Medical University Hospital (CSMUH No. CS1-25033) with a waiver of informed consent. All biospecimens and associated data were handled in an anonymized, de-linked manner.

### 2.2 PD-L1 immunohistochemistry

PD-L1 expression was assessed using the PD-L1 IHC 22C3 pharmDx assay (Agilent Technologies, Santa Clara, CA, USA) on the Dako Autostainer Link 48 platform, strictly following the manufacturer’s instructions (8, 9). Deparaffinized 4-micrometer FFPE sections underwent low-pH antigen retrieval and incubation with mouse monoclonal anti–PD-L1 antibody clone 22C3, followed by visualization with the EnVision FLEX detection system using diaminobenzidine (DAB) chromogen and hematoxylin counterstaining. Each immunohistochemical run included appropriate external positive and negative tissue controls, as well as PD-L1 control cell line slides, to ensure assay reliability.

### 2.3 Scoring and evaluation

Two board-certified pathologists (C.P.H. and W.R.C.) independently reviewed all slides to confirm diagnostic accuracy and specimen adequacy, including intact tissue and at least 100 viable tumor cells per sample according to the manufacturer’s criteria for the 22C3 pharmDx assay. Any discrepancies were resolved by consensus. The assay has been analytically and clinically validated for specific scoring systems and tumor indications, but not specifically for cervical NEC. TPS, TC, and CPS were recorded, and IC was assessed as an exploratory measure (10–15). Because final scores reflected consensus rather than independent scoring by each pathologist, a formal interobserver agreement statistic was not calculated; nonetheless, consensus scoring is expected to have reduced scoring variability relative to single-observer assessment.

Tumor Proportion Score (TPS): TPS was defined as the percentage of viable tumor cells showing partial or complete linear membranous PD-L1 staining of any intensity, excluding cytoplasmic-only staining, immune cells, and necrotic regions. TPS was recorded as an exploratory measure because no standardized TPS cutoff has been validated for cervical NEC.


$$\text{TPS}(\%)=\frac{PD‐L1(+)\text{ viable tumor cells}}{\text{Total viable tumor cells}}\times 100$$

Sun et al. used a TPS threshold of ≥1% for exploratory purposes in small-cell neuroendocrine carcinoma of the cervix; this threshold has not been validated as a clinical standard for cervical NEC (13).

Tumor Cell Score (TC): TC represents the percentage of viable tumor cells with membranous PD-L1 staining of any intensity.


$$\text{TC}(\%)=\frac{PD‐L1(+)\text{ viable tumor cells}}{\text{Total viable tumor cells}}\times 100$$

Because the TC and TPS definitions assessed the same tumor-cell membranous staining features in this study, the two scores were numerically equivalent in all six cases; TPS/TC is therefore reported jointly hereafter.

Combined Positive Score (CPS): CPS was calculated as the number of PD-L1–positive tumor cells, lymphocytes, and macrophages divided by the total number of viable tumor cells, multiplied by 100. Partial or complete membranous staining in tumor cells and membranous or cytoplasmic staining in mononuclear inflammatory cells within tumor nests or adjacent supporting stroma were included (8, 9).


$$\text{CPS}=\frac{PD‐L1(+)\text{ cells }(\text{tumor cells},\text{ lymphocytes},\text{ macrophages})}{\text{Total viable tumor cells}}\times 100$$

According to the PD-L1 IHC 22C3 pharmDx Interpretation Manual for cervical cancer, CPS ≥1 defines PD-L1 expression, and neutrophils, eosinophils, and plasma cells are excluded from the CPS numerator (8, 9). In the present study, this scoring approach was applied to cervical NEC as an investigational method rather than as a histology-specific validated standard.

Immune Cell (IC) score: IC was defined as the percentage of tumor area occupied by PD-L1-positive immune cells (lymphocytes and macrophages) within intratumoral nests and adjacent stroma, excluding stromal fibroblasts, benign epithelium, plasma cells, and vascular elements.


$$\text{IC}(\%)=\frac{Tumor area infiltrated by PD‐L1(+)\text{ immune cells}}{\text{Total tumor area}}\times 100$$

In the absence of a validated IC threshold for the 22C3 assay or for cervical NEC, the exploratory ≥5% reference reported by Sun et al. (13) was applied for descriptive comparison; the rationale for this choice and its methodological limitations are detailed in the Discussion (§4.2).

### 2.4 Statistical analysis

Given the small cohort (n = 6), all data were analyzed descriptively; no inferential statistical methods were applied. The study was not designed or powered for survival analysis or for evaluating associations between PD-L1 scores and clinicopathologic or clinical outcome variables.

## 3 Results

### 3.1 Cohort characteristics and overall PD-L1 expression

Among the six cervical NEC cases, two were classified as small-cell neuroendocrine carcinoma (SCNEC) and four as large-cell neuroendocrine carcinoma (LCNEC). PD-L1 scores assessed using the 22C3 pharmDx assay are summarized in **Table 1**.

**Table 1:** PD-L1 expression (22C3 assay) scores in cervical neuroendocrine carcinomas.

Case no.	Histological subtype	TPS (%)	TC (%)	CPS (%)	IC (%)
1	SCNEC	0	0	<1	<1
2	LCNEC	<1	<1	5	5
3	LCNEC	0	0	3	10
4	SCNEC	3	3	25	30
5	SCNEC	0	0	0	0
6	SCNEC	0	0	0	0

1. SCNEC, small-cell neuroendocrine carcinoma; LCNEC, large-cell neuroendocrine carcinoma; TPS, Tumor Proportion Score; TC, Tumor Cell Score; CPS, Combined Positive Score; IC, Immune Cell Score.

2. TC values were identical to TPS values in all six cases; both columns are presented for transparency.

### 3.2 Tumor-cell PD-L1 expression by TPS/TC

By TPS/TC, membranous PD-L1 was detected in tumor cells in Case 4 (3%) and at a marginal level in Case 2 (<1%); the remaining four cases showed no detectable tumor-cell staining.

### 3.3 Combined positive score findings

CPS, which incorporates both tumor-cell and immune-cell staining, ranged from <1 to 25. Case 4 had the highest CPS (25), followed by Case 2 (5) and Case 3 (3); Case 1 had a CPS <1, whereas Cases 5 and 6 had CPS = 0. The high CPS in Case 4 was accompanied by prominent immune-cell staining.

### 3.4 Immune cell score findings

IC scoring likewise showed variable PD-L1 expression among immune infiltrates: 30% in Case 4, 10% in Case 3, 5% in Case 2, <1% in Case 1, and 0% in Cases 5 and 6.

### 3.5 Integrated PD-L1 distribution and representative findings

Overall, CPS ≥1 and IC ≥5% were each observed in three of six tumors (50%), whereas TPS/TC ≥1% was observed in one case (16.7%) (**Table 1**). PD-L1 staining was more frequently localized to the immune-cell compartment than to tumor cells in this cohort.

**Figure 1** shows representative histopathology of Case 4. H&E staining illustrates characteristic NEC morphology, and immunohistochemistry demonstrated diffuse synaptophysin, CD56, and nuclear INSM1 expression. PD-L1 staining showed heterogeneous membranous expression in a subset of tumor cells and intense staining in immune cells within and around tumor nests, corresponding to the highest scores in the cohort (CPS 25, TPS/TC 3%, IC 30%).

**Figure 1:**
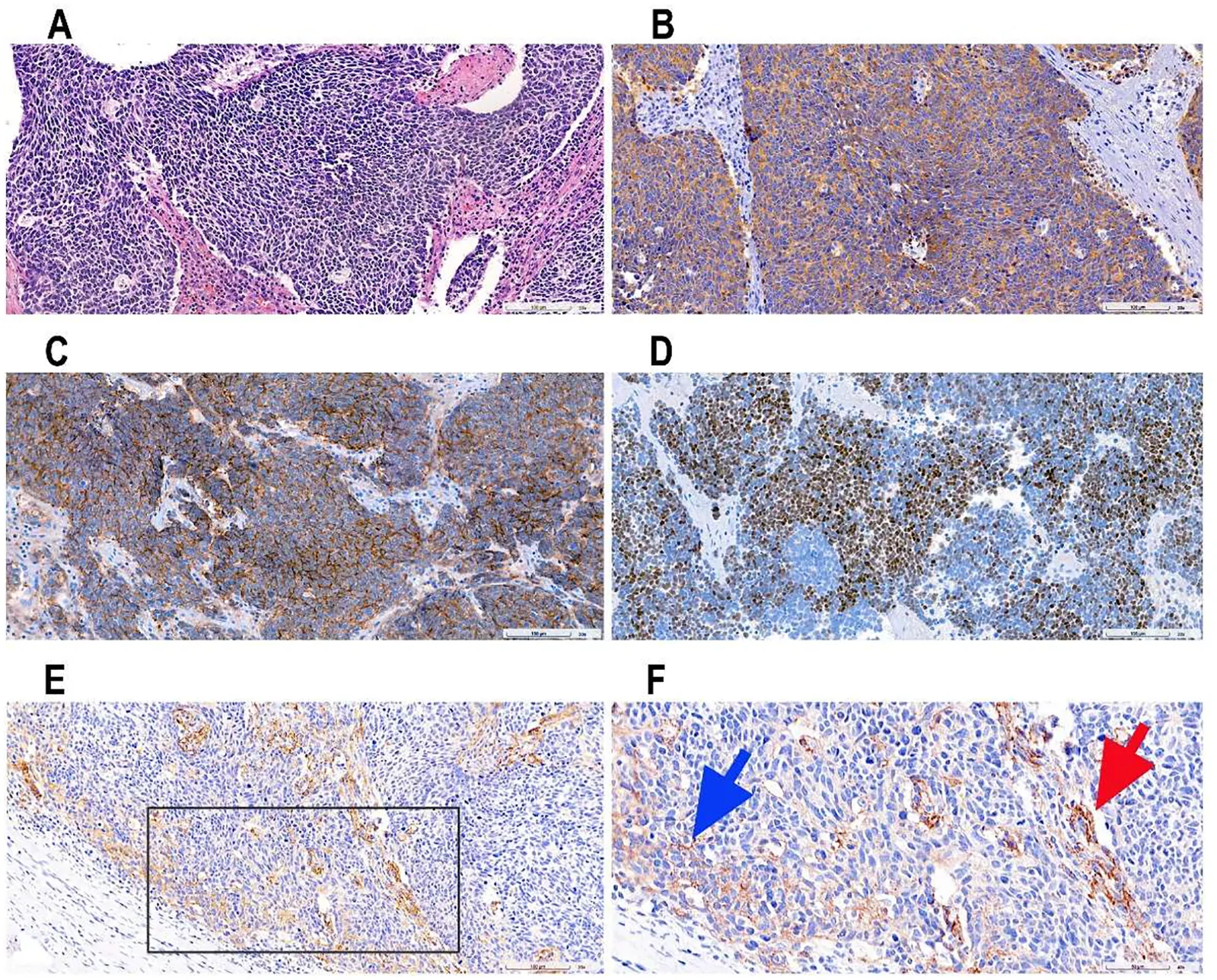
Representative case (case No. 4) of cervical neuroendocrine carcinoma (NEC). **(A)** Hematoxylin and eosin (H&E) staining reveals tumor cells with scant cytoplasm, hyperchromatic nuclei, nuclear molding, and finely granular chromatin, predominantly arranged in solid sheets with focal rosette formation (200×). **(B)** Synaptophysin immunostaining reveals diffuse cytoplasmic and membranous expression (200×). **(C)** CD56 immunostaining demonstrates diffuse cytoplasmic and membranous positivity (200×). **(D)** INSM1 shows strong nuclear positivity in tumor cells (200×). **(E)** PD-L1 immunohistochemistry using the PD-L1 IHC 22C3 pharmDx assay shows heterogeneous, positive staining in both tumor cells and immune cells at low magnification (200×); the boxed area is enlarged in panel **(F)** at high magnification (400×). **(F)** The blue arrow indicates tumor cells with partial and variable membranous PD-L1 expression. In contrast, the red arrow highlights intense PD-L1 staining in the immune cells located within and immediately adjacent to tumor nests. The PD-L1 CPS is 25, the TPS/TC score is 3%, and the IC score is 30%.

## 4 Discussion

### 4.1 Clinical context, rationale, and study contribution

Cervical NEC is a rare and clinically aggressive malignancy for which disease-specific treatment strategies remain limited. Although PD-L1 assessment may provide information about the tumor immune microenvironment, its clinical interpretation in cervical NEC remains uncertain because the relevant PD-L1 assays and scoring thresholds have not been specifically validated for this histologic subtype. Within this context, the principal contribution of this study is its assay-specific, descriptive characterization of PD-L1 distribution across tumor-cell and immune-cell compartments in cervical NEC, using the PD-L1 IHC 22C3 pharmDx assay, the FDA-approved companion diagnostic for pembrolizumab-eligibility testing, with TPS/TC, CPS, and IC assessed in parallel.

### 4.2 Assay-specific validation and scoring considerations

PD-L1 scoring systems, including TPS/TC, CPS, and IC, are assay- and indication-specific, and their thresholds should not be extrapolated across tumor types or therapeutic settings. In cervical cancer, PD-L1 IHC 22C3 pharmDx uses CPS ≥1 as an approved criterion for pembrolizumab treatment; however, this criterion has not been validated for cervical NEC (8, 9). Similarly, an IC cutoff of ≥1% is an assay-specific threshold established with the Ventana PD-L1 SP142 assay for metastatic triple-negative breast cancer (15). Neither threshold has been validated for cervical NEC, and the thresholds used in this study were therefore investigational and descriptive rather than histology-specific clinical standards.

Because no IC threshold specific to the 22C3 assay or cervical NEC has been established, the ≥5% threshold reported by Sun et al. was used only as an exploratory reference (13). Histologic relevance was prioritized over assay concordance: although the SP142 IC score shares our area-based scoring convention, it was validated in the biologically distinct triple-negative breast cancer, whereas the Sun et al. threshold was derived specifically from cervical NEC. However, the IC measure used in this study and the ICS reported by Sun et al. were derived using different assay platforms, antibody clones, and scoring procedures. Our IC score was defined as the percentage of tumor area occupied by PD-L1-positive immune cells, as measured by the 22C3 assay. In contrast, Sun et al. assessed ICS using the SP263 clone and study-specific criteria (13). Therefore, the two measures and their thresholds are not directly comparable, and the ≥5% threshold was used only for descriptive comparison rather than as a validated or regulatory-endorsed standard for cervical NEC.

TPS/TC, CPS, and IC were therefore interpreted only as descriptive or exploratory measures in this six-case cohort. Given the immune-cell–predominant staining pattern, IC scoring may provide complementary information for future research; however, its clinical and predictive relevance in cervical NEC remains uncertain and requires validation in larger studies that integrate functional immune profiling with clinical outcomes.

### 4.3 Patterns of PD-L1 expression: cohort and literature comparison

In this cohort, PD-L1 staining was more frequently detected in the immune-cell compartment than in tumor cells. CPS ≥1 and IC ≥5% were each observed in three of six cases (50%), with Case 4 showing the highest values (CPS 25; IC 30%), whereas TPS/TC ≥1% was observed only in Case 4 (3%) (**Table 1**). These findings are within the range reported in previous cervical NEC series, although differences in antibody clones, scoring methods, cutoff values, and cohort sizes limit direct comparison (13, 16–21). Reported CPS-based positivity rates have ranged from approximately 8% to 70%, while Sun et al. reported TPS-, CPS-, and IC-defined positivity in 59.6%, 68.5%, and 33.7% of tumors, respectively (**Table 2**). The immune-cell-predominant pattern observed in the present cohort is descriptive, and its biological and clinical significance cannot be determined from these data alone.

**Table 2:** Reports of PD-L1 expression in cervical NEC.

Author [ref]	Year	Total case number	Positive case number (%)	Assay method/cut-off	PD-L1 antibody used
Morgan et al. (16)	2019	10	7 (70.0%)	H-score ≥10*	Ventana, clone SP263
Carroll et al. (17)	2020	25	2 (8.0%)	CPS≥1	Dako Agilent, clone 22C3
Ji et al. (18)	2021	20	14 (70.0%)	CPS≥1	Abcam, clone ab205921
Cimic et al. (19)	2021	39	4 (10%)	CPS≥1	Dako Agilent, clone 22C3
Sun et al. (13)	2022	89	53 (59.6%) 30 (33.7%) 61 (68.5%)	TPS≥1% ICS≥5%**^§^** CPS≥1	Ventana, clone SP263
Li et al. (20)	2023	45	17 (37.8%)	CPS≥1	Dako Agilent, clone 28-8
Qiu et al. (21)	2024	29	6 (20.7%)	CPS≥1	Dako Agilent, clone 22C3
Our cohort	2026	6	1 (16.7%) 3 (50.0%) 3 (50.0%)	TPS/TC≥1% IC≥5%^§^ CPS≥1	Dako Agilent, clone 22C3

*The PD-L1 H-score in this study was a semi-quantitative IHC measure based on staining intensity and the percentage of tumor cells. An H-score >10 was considered positive in Morgan et al.; this cutoff was specific to that study and is not a universal standard (16).

^§^ Sun et al. considered an IC score ≥5% positive. The same exploratory threshold was applied in the present study (13).

### 4.4 Biological and therapeutic implications

PD-L1 expression within the tumor microenvironment may reflect multiple regulatory processes. Tumor-cell PD-L1 expression can arise through intrinsic signaling pathways, whereas immune-cell PD-L1 expression may reflect adaptive responses to local inflammatory signals, including interferon-γ-mediated induction. These mechanisms provide a literature-based framework for interpreting PD-L1 staining across cellular compartments (22–26). However, cytokine signaling, T-cell function, and immune-cell composition were not directly assessed in this study; therefore, these mechanisms should be regarded as contextual interpretations rather than findings demonstrated in this cohort.

Although cervical NEC has been associated with high-risk HPV infection (27), the effects of HPV-driven oncogenesis on the cervical immune microenvironment were not directly assessed in our six-case cohort. Careful examination of the surrounding cervix identified no associated precursor lesions, including high-grade squamous intraepithelial lesion (HSIL) or adenocarcinoma *in situ* (AIS). The relationship between HPV-related oncogenesis and the cervical immune microenvironment warrants further investigation.

**Figure 2** illustrates the PD-1/PD-L1 pathway and its therapeutic blockade as conceptual background for interpreting the immunohistochemical findings. Although PD-1/PD-L1 blockade has shown clinical benefit in selected tumor types, its relevance to cervical NEC remains uncertain. The present PD-L1 IHC findings alone do not establish an immune phenotype, treatment susceptibility, or therapeutic benefit (28–31).

**Figure 2:**
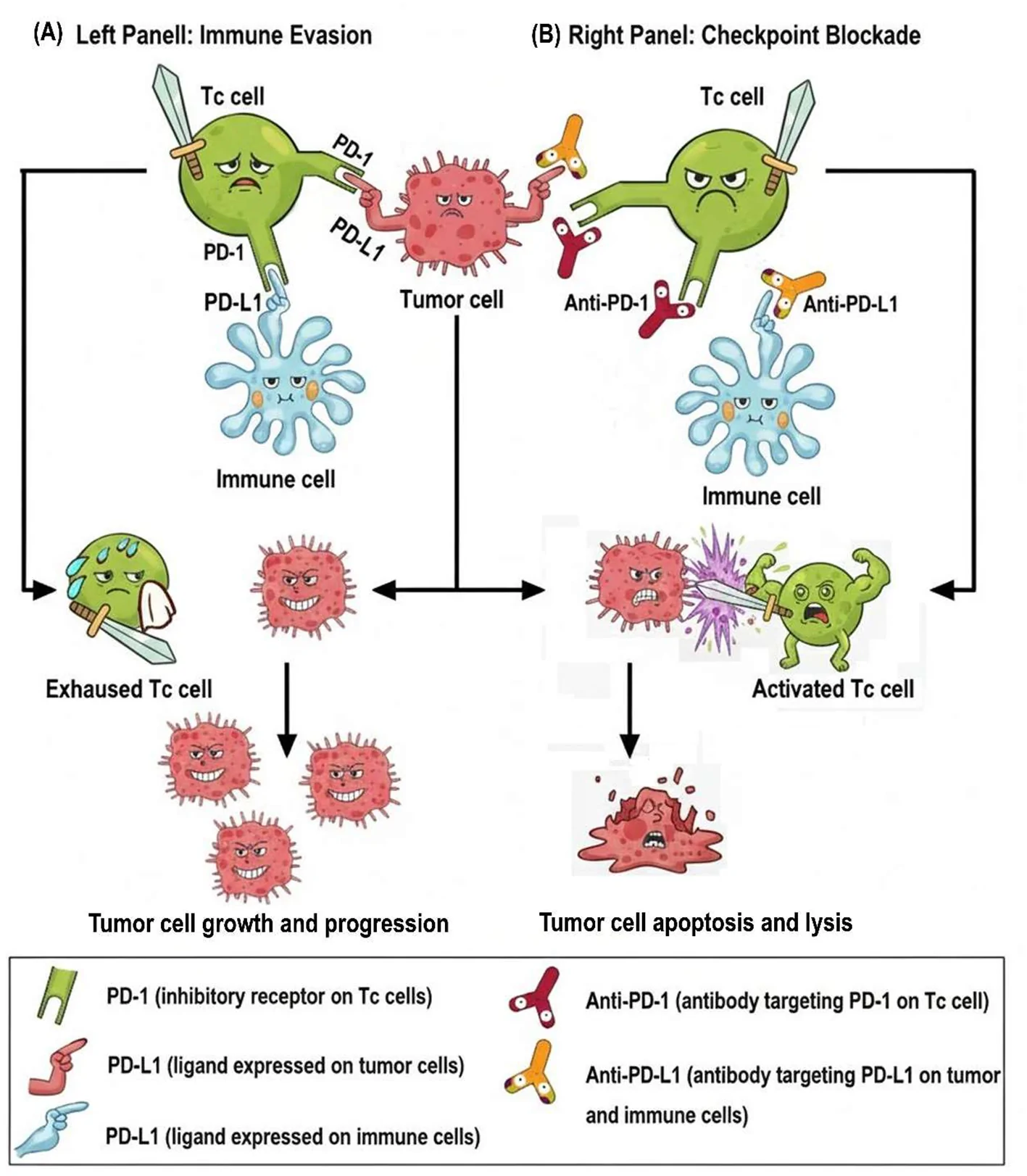
PD-1/PD-L1–mediated immune evasion and therapeutic checkpoint blockade. **(A)** Immune evasion (left panel). Tumor cells evade immune surveillance through the PD-1/PD-L1 pathway. PD-L1 expressed on tumor cells (via intrinsic oncogenic signaling) and on tumor-associated immune cells (via adaptive induction) binds to PD-1 on cytotoxic T lymphocytes (CTLs). This inhibitory interaction suppresses T-cell activation and effector function, leading to T-cell exhaustion and permitting sustained tumor growth and progression. **(B)** Immune checkpoint blockade (right panel). Therapeutic antibodies targeting PD-1 or PD-L1 disrupt this inhibitory interaction. Blockade of PD-1/PD-L1 signaling restores CTL activation and cytotoxic function, leading to tumor cell apoptosis and lysis, thereby enhancing antitumor immunity.

Case 4 showed concurrent PD-L1 staining in tumor cells and tumor-infiltrating immune cells (**Figures 1E, F**), illustrating the heterogeneity of PD-L1 distribution. Because this pattern was observed in a single case without functional or clinical outcome data, its biological and therapeutic significance remains uncertain.

### 4.5 Limitations

The small cohort size, together with the anonymized, de-linked design mandated by the IRB-approved waiver of consent, precluded retrospective re-linkage to individual clinical records and thus limited clinicopathologic correlation and reliable reconstruction of treatment history, follow-up, and survival outcomes. Because the study was designed as a descriptive, assay-focused PD-L1 analysis of archival FFPE tissue, functional immune characterization was not feasible.

### 4.6 Future directions

Prospective, multicenter studies integrating linked clinical outcomes, HPV and p16 status, genomic profiling, and functional immune characterization are warranted to clarify the biological and clinical relevance of PD-L1 expression, particularly within the immune-cell compartment, in cervical NEC.

## 5 Conclusion

Given the small sample size, these preliminary findings are descriptive, exploratory, and hypothesis-generating rather than definitive. In this cohort, PD-L1 expression was heterogeneous. Tumor-cell positivity by TPS/TC was uncommon, occurring in a single case, whereas CPS and IC more frequently identified PD-L1-positive cases, reflecting the predominance of immune-cell staining. However, the biological and clinical significance of this distribution remains uncertain, and the predictive utility of CPS and IC for cervical NEC has not been validated. Larger, multicenter studies integrating molecular profiling, functional immune characterization, and clinical outcomes are needed to determine whether PD-L1 assessment has clinical utility in this rare malignancy.

## Data Availability

The original contributions presented in the study are included in the article. Further inquiries can be directed to the corresponding authors.
